# Global Prioritizing Disease Candidate lncRNAs via a Multi-level Composite Network

**DOI:** 10.1038/srep39516

**Published:** 2017-01-04

**Authors:** Qianlan Yao, Leilei Wu, Jia Li, Li guang Yang, Yidi Sun, Zhen Li, Sheng He, Fangyoumin Feng, Hong Li, Yixue Li

**Affiliations:** 1 School of Life Sciences and Biotechnology, Shanghai Jiao Tong University, Shanghai, 200031, China; 2 CAS Key Laboratory for Computational Biology, CAS-MPG Partner Institute for Computational Biology, Shanghai Institute for Biological Sciences, Chinese Academy of Sciences, Shanghai, 200031, China; 3 University of Chinese Academy of Sciences, Beijing, 100049, China; 4 Collaborative Innovation Center of Genetics and Development, Fudan University, Shanghai 200433, China

## Abstract

LncRNAs play pivotal roles in many important biological processes, but research on the functions of lncRNAs in human disease is still in its infancy. Therefore, it is urgent to prioritize lncRNAs that are potentially associated with diseases. In this work, we developed a novel algorithm, LncPriCNet, that uses a multi-level composite network to prioritize candidate lncRNAs associated with diseases. By integrating genes, lncRNAs, phenotypes and their associations, LncPriCNet achieves an overall performance superior to that of previous methods, with high AUC values of up to 0.93. Notably, LncPriCNet still performs well when information on known disease lncRNAs is lacking. When applied to breast cancer, LncPriCNet identified known breast cancer-related lncRNAs, revealed novel lncRNA candidates and inferred their functions via pathway analysis. We further constructed the human disease-lncRNA landscape, revealed the modularity of the disease-lncRNA network and identified several lncRNA hotspots. In summary, LncPriCNet is a useful tool for prioritizing disease-related lncRNAs and may facilitate understanding of the molecular mechanisms of human disease at the lncRNA level.

Recent studies have revealed that up to 70% of the human genome is transcribed into RNA, whereas protein-coding genes only make up less than 2% of the total genome. The majority of the transcriptional repertoire consists of non-coding RNAs (ncRNAs)^1^,^2^. Long non-coding RNAs (lncRNAs), which constitute the majority of ncRNAs, are a class of transcripts longer than 200 nt that lack protein-coding potential^3^,^4^. Accumulating evidence indicates that lncRNAs play pivotal roles in many important biological processes^5^,^6^. In particular, recent studies have suggested that lncRNAs are involved in the initiation and progression of a wide range of diseases^7^ and have been found to act as tumor suppressors or oncogenes^8^. For example, the lncRNA HOTAIR is dysregulated in several cancers, including colon, breast, pancreas, and liver cancers, and the overexpression of HOTAIR has been shown to drive breast cancer metastasis^9^. Therefore, identifying potential disease lncRNAs may facilitate understanding of the molecular mechanisms of human disease at the lncRNA level and may unveil new diagnostic and therapeutic opportunities. RNA-seq and microarray technologies have identified tens of thousands of human lncRNAs, but knowledge of disease-related lncRNAs is still limited. Therefore, it is a challenging task to prioritize lncRNAs associated with a high risk of disease for further functional investigation.

Recently, some computational methods have been proposed to predict disease-related lncRNAs. Some of these methods are based on the sequence or genomic locations of lncRNAs^10^,^11^,^12^. For example, a global disease-lncRNA associations have been predicted by LncRNADisease based on lncRNAs and their genomic loci related neighbor genes/miRNAs (within 2 kb) tend to be associated with the same disease^11^. A sequence based bioinformatics tool was proposed to predict lncRNA-disease associations using their interaction with disease-related miRNA^12^. LncRNA-mRNA co-expression-based methods have also been widely used to predict lncRNA function^13^,^14^ and to predict disease lncRNAs^15^. On the basis of the assumption that similar diseases tend to be associated with similar functional lncRNAs, several network-based methods have been developed to prioritize disease-related lncRNAs^16^,^17^,^18^,^19^. Sun *et al*. have presented the RLncD method using random walking on a lncRNA functional similarity network^18^. Zhou *et al*. have developed the RWRHLD method, involving random walking on a heterogeneous network integrating phenotype and disease information^17^,^19^. Most recently, IRWRLDA has been developed by integrating lncRNA expression similarity and disease semantic similarity, thereby effectively improving the prediction power^20^. On the basis of these previous results, we believe that associations between lncRNAs and genes, and disease-gene information are valuable information, and should be integrated in the study of lncRNA function. This integration is a feature unique to LncPriCNet.

It is reasonable to integrate multi-level data regarding genes, phenotypes, lncRNAs and their association information to prioritize disease-related lncRNA candidates. First, from a biological perspective, lncRNAs rarely function in isolation; instead, lncRNAs serve as regulators that may achieve regulatory specificity through modularity, assembling diverse combinations of proteins and possibly RNA and DNA interactions^21^. Some studies have suggested that lncRNAs may act synergistically^22^,^23^. Thus, a biological system can be intrinsically represented as an intricate network including multi-level information. The effects of one disease are not restricted to one or two lncRNAs but instead are spread among functionally related lncRNAs and genes. An integration strategy may provide more comprehensive and accurate information^24^. Second, although some experimentally determined disease-lncRNA associations have been collected^11^, completeness remains a distant goal. The integration strategy could use other information (such as disease-gene information and lncRNA-gene associations) to compensate for some missing information. Third, although it is important to investigate the functions of lncRNAs and uncover the mechanisms of biological processes, the functions of most lncRNAs remain unknown^7^. An integration strategy uses information about the genes associated with lncRNAs to facilitate interpretation. This integration strategy has been successfully used to disease metabolites prioritization and improve the prediction power^25^.

Assuming that functionally related lncRNAs and genes play roles in phenotypically similar diseases, we proposed a computational method called LncPriCNet (disease candidate LncRNAs Prioritization based on a Composite Network) to prioritize disease-related lncRNAs. First, we constructed a composite network integrating multi-level information including phenotypes, lncRNAs, genes and their associations. Fully considering the global functional interactions of the multi-level composite network, LncPriCNet prioritizes the candidate lncRNAs on the basis of their similarity to known disease information. LncPriCNet has better predictive power than previous methods. Importantly, by integrating multi-level information, LncPriCNet performs well even for diseases without known associated lncRNAs. When applied to a breast cancer RNA-Seq data set, LncPriCNet identified known disease-related lncRNAs as well as novel ones. Furthermore, a disease-lncRNA landscape was constructed and analyzed to provide a global view of disease lncRNAs.

## Materials and Methods

### Data sources and construction of a multi-level composite network

**Figure 1:**
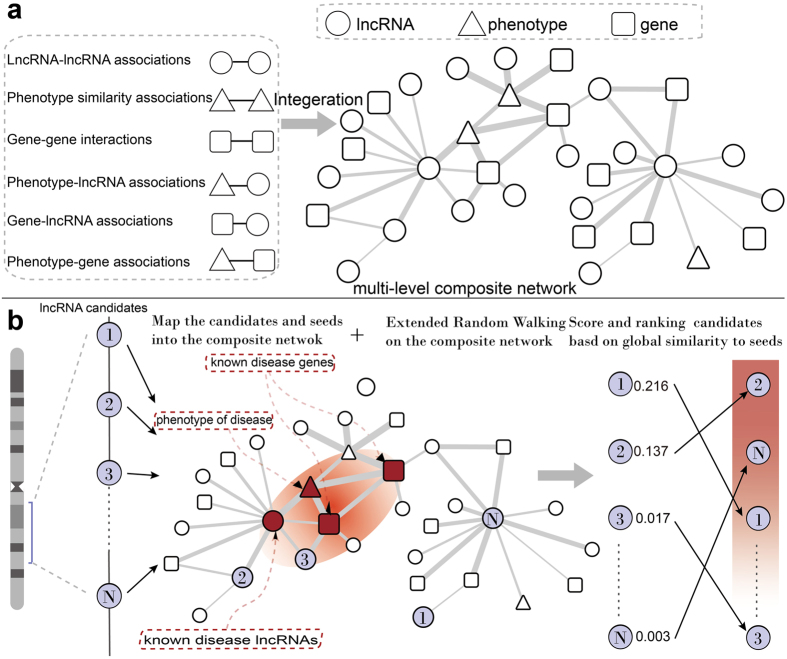
The flow chart of LncPriCNet. (**a**) Construction of the multi-level composite network. This network is constructed by six sub-networks. White circle indicates lncRNA; white square indicates gene; white triangle indicates phenotype. The thickness of the edge indicates the weight score. (**b**) The flow chart by which LncPriCNet optimizes the candidate lncRNAs. First, the candidate lncRNAs of interest and seed nodes are mapped to the multi-level composite network. Then, a global extended RWR method is used to score the candidate lncRNAs according to their proximity to seed nodes. Finally, the candidate lncRNAs are ranked according to the scores. Purple circles represent the candidate lncRNAs of interest; red triangle indicates disease phenotype (phenotype seed) of interest from the OMIM data base; red squares represent known disease genes (gene seeds) from the OMIM database; and red circles indicate known disease lncRNAs (lncRNA seeds) from the lncRNADisease database.

To construct a multi-level composite network, we collected experimentally validated or computationally predicted associations among phenotypes, genes and lncRNAs (Fig. 1a). The gene-gene associations were constructed on the basis of protein-protein interaction data downloaded from the HPRD database^26^. The disease-phenotype associations were obtained from the OMIM database^27^ by removing records with the prefixes “*” and “^”. The textual similarity between disease phenotypes was calculated by MimMiner^28^. The top five similar phenotypes were retained for analysis. RNA-seq data on 16 human tissues were obtained from the Human Body Map project (ERP000546). We used Tophat^29^ to perform read alignment and cufflinks^30^ to perform transcript assembly, and the expression levels of lncRNAs and coding genes were estimated as FPKM (fragments per kilobase of transcript per million fragments mapped). LncRNA-lncRNA and gene-lncRNA associations were measured using the Pearson correlation coefficient. The experimentally verified gene-lncRNA associations were also obtained if they were supported by more than two CLIP-seq experiments in the StarBase database^31^. Experimentally verified disease-lncRNA relationships were taken from the lncRNADisease database^11^. After removal of redundant records, 371 disease-lncRNA relations between 108 lncRNAs and 140 disease phenotypes were obtained. The phenotype-gene relations were extracted from the OMIM database by BioMart^32^.

Next, we integrated the above information to construct a weighted multi-level composite network. The edge weights were defined on the basis of the data sources: 1 for experimentally validated associations and correlation coefficients for computationally predicted associations. Let *W*_*G*_, *W*_*P*_, *W*_*L*_, *W*_*GL*_, *W*_*PL*_, *W*_*GP*_ be the adjacency matrix of the gene network, phenotype network, lncRNA network, gene-lncRNA network, phenotype-lncRNA network and gene-phenotype network, respectively. Then, the adjacency matrix of the multi-level composite network can be defined as 
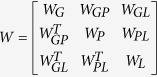
, where the superscript T means the transpose of matrix.

### LncPriCNet

We propose a novel global computational method to prioritize disease-related lncRNA (LncPriCNet), which extends the random walking with restart (RWR) algorithm to a multi-level network to capture global information (Fig. 1b). It simulates a random walker walking on the network, starting on a set of seed nodes, and at each step, it moves from the current nodes to their direct neighbor(s) randomly with a probability 1 − *δ*, then returns to the seed nodes with a restart probability *δ*. Let *P*^0^ be the initial probability vector, and let *P*^*t*^ represent a vector in which the *i-th* element is the probability of being at node *i* at step *t*. Then, the probability vector at step *t* + 1 is defined as follows:


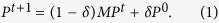


where *M* is the transition matrix of the multi-level composite network, which can be calculated by the adjacency matrix *W* (the computational details will be described later). After several iterations, the probability will reach a steady state when the difference between *P*^0^ and *P*^*t*^ falls below 10^−10^ (measured by the L1 norm).

For one disease-phenotype of interest, the seed nodes defined in this study consist of this disease-phenotype (S^P^), and its corresponding known disease gene (S^G^) and lncRNA (S^L^). Suppose *u*_0_, *v*_0_, *w*_0_ are the initial probabilities of the gene network, phenotype network and lncRNA network, respectively. Here, *u*_0_ is calculated by assigning equal probability to all nodes in S^G^ in the gene network with a sum equal to 1. Similarly, *v*_0_, *w*_0_ can be calculated. Then, the initial probability vector of the multi-level composite network is denoted as 
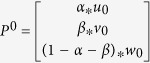
. Here, *α, β* and 1 − *α* − *β* range from 0 to 1, and they represent the importance of the gene network, phenotype network and lncRNA network, respectively.

Let the transition matrix 
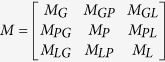
. *M*_*G*_, *M*_*P*_, *M*_*L*_ denote the intra-subnetwork transition matrix, and other variables denote the inter-subnetwork transition matrix. *M*_*ij*_ represents the transition probability from node *i* to node *j*. Suppose *x, y, z* are the jumping probability between the gene network and phenotype network, between the gene network and lncRNA network, and between the phenotype network and lncRNA network or vice versa, respectively. Then, the transition probability from gene *i* (*g*_*i*_) to gene *j* (*g*_*j*_) in the gene network can be computed as follows:


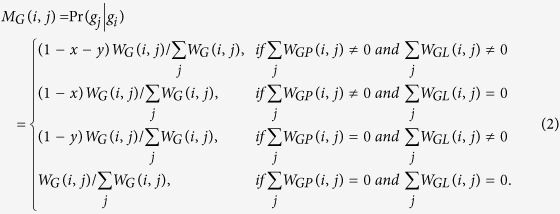


Similarly, the transition probability from phenotype *i* (*p*_*i*_) to phenotype *j* (*p*_*j*_) in the phenotype network can be calculated as follows:


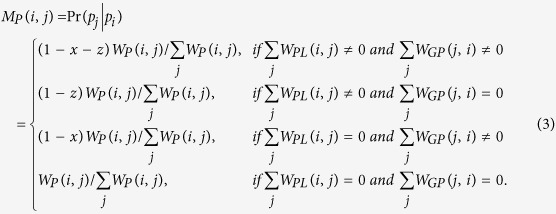


The probability from lncRNA *i* (*l*_*i*_) to lncRNA *j* (*l*_*j*_) in the lncRNA network can be computed as


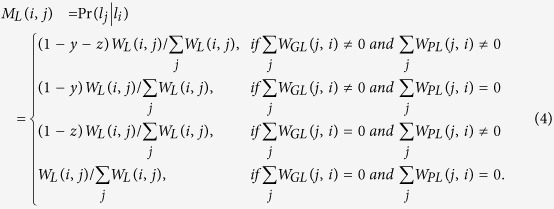


The transition probability from gene *i* (*g*_*i*_) in the gene network to phenotype *j* (*p*_*j*_) in the phenotype network can be defined as


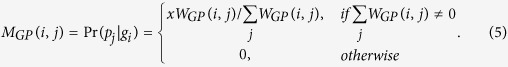


The transition probability from gene *i* (*g*_*i*_) in the gene network to lncRNA *j* (*l*_*j*_) in the lncRNA network can be described as


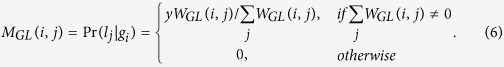


The transition probability from phenotype *i* (*p*_*i*_) in the phenotype network to gene *j* (*g*_*j*_) in the gene network can be defined as


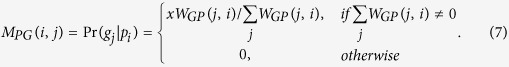


The transition probability from phenotype *i* (*p*_*i*_) to lncRNA *j* (*l*_*j*_) can be described as


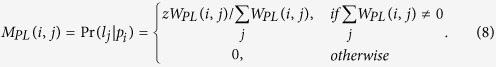


The transition probability from lncRNA *i* (*l*_*i*_) to gene *j* (*g*_*j*_) can be defined as


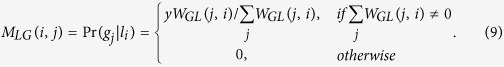


The transition probability from lncRNA *i* (*l*_*i*_) to phenotype *j* (*p*_*j*_) can be described as


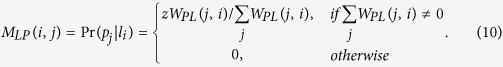


To further describe the equations for the transition probability, we take equation (10) as an example. Suppose the current node is a lncRNA *i* (*l*_*i*_), and *M*_*LP*_ (*i, j*) denotes the probability of moving from this lncRNA *i* (*l*_*i*_) to a phenotype node *j* (*p*_*j*_). In this step, 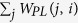 means that no phenotypes directly link to this lncRNA, and thus the walker never moves to a phenotype node in this step, and the probability should be 0. Otherwise, 
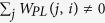
 means the walker could move to one of phenotype nodes. Then, the probability that the walker moves from this lncRNA *i* (*l*_*i*_) to phenotype *j* (*p*_*j*_) should be calculated by multiplying the jumping probability *z* by a normalized adjacency matrix of the phenotype-lncRNA network 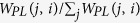.

LncPriCNet is performed until the probabilities tend to a steady state, 
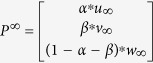
. Then, the candidate lncRNAs can be ranked according to *w*_∞_. In this study, we set the parameter δ to 0.7 and *x, y, z, α, β* to 1/3.

LncPriCNet is implemented in the R language. An R-based package of LncPriCNet is available at https://cran.r-project.org/.

## Results

We first constructed a multi-level composite network integrating information on lncRNAs, genes, phenotypes and their associations (Fig. 1a). This network consisted of three types of node (gene, lncRNA and phenotype) and six types of association (gene-gene, lncRNA-lncRNA, phenotype-phenotype, phenotype-lncRNA, phenotype-gene and gene-lncRNA) (Table S1). Then, we developed a novel global computational method to prioritize disease-related lncRNAs on the basis of this multi-level composite network (LncPriCNet) (Fig. 1b). In this section, we first evaluated the performance of LncPriCNet and compared it with other methods. Then, we applied LncPriCNet to a breast cancer dataset to confirm its ability to find novel disease lncRNA candidates. Furthermore, a disease-lncRNA landscape was constructed and analyzed to provide a global view of disease-related lncRNAs.

### Performance of LncPriCNet

**Figure 2:**
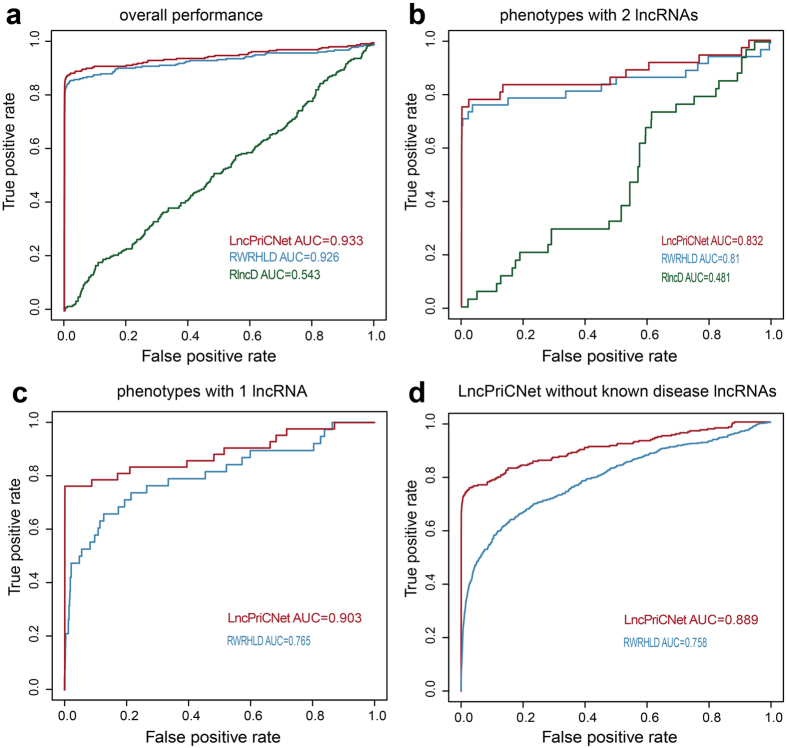
Performance of LncPriCNet and comparison with other methods. (**a**) ROC curve for the predicted lncRNAs of 53 phenotypes. (**b**) ROC curve for the predicted lncRNAs of 20 phenotypes with two known lncRNAs. (**c**) ROC curve for the predicted lncRNAs of 42 phenotypes with only one known lncRNA. (**d**) The performance in hypothetical phenotypes without known disease lncRNAs.

To test the performance of LncPriCNet, we used leave-one-out cross validation (LOOCV) to assess whether LncPriCNet could identify known disease lncRNAs. First, we chose 53 phenotypes associated with at least two experimentally validated lncRNAs in the composite network. We obtained 284 known phenotype-lncRNA and 153 known phenotype-gene associations. For each round of cross-validation, one known disease lncRNA was selected as a test object, and links between this lncRNA and its corresponding target phenotype were removed. We defined the seed nodes as the target phenotype, other known lncRNAs associated with this phenotype and all known genes associated with this phenotype. Then, LncPriCNet was performed and returned a score for each non-seed lncRNA. The top-ranked lncRNAs were predicted to be related to the disease. Receiver operating characteristic analysis (ROC) and the area under the curve (AUC) were used to evaluate the overall performance of LncPriCNet. ROC was performed by plotting the true positive rate versus the false positive rate at various score threshold settings. The results showed that LncPriCNet achieved an AUC value up to 0.933 (Fig. 2a). Additionally, over 88.3% (251) of known disease-related lncRNAs were ranked in the top 10%, and even in the top 10, there were still 74.3% known disease-related lncRNAs. These results suggested that our strategy of using multi-level data from the composite network is effective in prioritizing disease lncRNAs.

To further investigate the performance of LncPriCNet in different disease classes, 53 phenotypes were grouped into 10 disease classes on the basis of previous work^33^ and manually annotated. LOOCV was performed for each disease class, and the AUC value was calculated. LncPriCNet achieved an AUC value over 0.7 in 8 disease classes, and in 4 classes, the AUC value was over 0.93 (Table S2). The metabolic disease class ranked in the top 1 (AUC = 1), and all six known metabolic lncRNAs were ranked in the top 3 by LOOCV. The cardiovascular disease class ranked in the top 2 (AUC = 0.999), and 68 (98.5%) known cardiovascular lncRNAs were ranked in the top 2 by LOOCV.

### Method comparison

To highlight the superiority of integrating multi-level information, we compared LncPriCNet with RWRHLD and RlncD. All the three methods use random walking with restart on a network; LncPriCNet uses the phenotype-lncRNA-gene network, whereas RWRHLD uses the phenotype-lncRNA network^19^, and RlncD uses the lncRNA network^18^. The AUC values of RWRHLD and RlncD were 0.926 and 0.543, which was lower than that of LncPriCNet (Fig. 2a). When performed on different disease classes, LncPriCNet resulted in higher AUC values than the other two methods in 7 of 10 (70%) disease classes (Fig. S1 and Table S2).

Because the known lncRNA-disease associations remain limited, we further evaluated the performance of three methods for diseases lacking known lncRNAs. First, we extracted 20 phenotypes linked to only two known lncRNAs, after which 40 known disease lncRNAs and 40 known disease genes remained. The ROC curve obtained from LOOCV showed that LncPriCNet performed better than RWRHLD and RlncD (Fig. 2b). Second, we extracted 42 phenotypes with only one known disease lncRNA in the composite network, and we obtained 42 known lncRNAs and 71 known genes. As shown in Fig. 2c, LncPriCNet achieved an AUC value of 0.903, which was much higher than that of RWRHLD (AUC value of 0.765). Third, we assumed that all 53 phenotypes were linked to no known disease lncRNAs and performed LOOCV. For each run of LOOCV, all links between the phenotype and known lncRNAs were removed, and then the phenotype and known disease genes were used as seeds to recall the known lncRNAs. As shown in Fig. 2d, LncPriCNet achieved an AUC value of 0.889, whereas RWRHLD obtained an AUC value of only 0.758. RlncD completely lost efficiency under the last two conditions. These results showed that LncPriCNet performs better than the previous methods, especially for diseases with few known lncRNAs.

### Parameters of LncPriCNet

LncPriCNet has five parameters: δ, *x, y, z, α* and *β*. Here, δ is the restart probability in the random walk method, and *x, y, z* are the jumping probability between gene network and phenotype network, between the gene network and lncRNA network, and between the phenotype network and lncRNA network or vice versa. The values of *α, β* and 1 − *α* − *β* range from 0 to 1, and they represent the importance of the gene network, phenotype network and lncRNA network, respectively. To investigate the possible effects of these parameters, different values were assigned to these parameters and LOOCV analysis was performed. The resulting AUC values varied from 0.879 to 0.943 (Tables S3 and S4), thus suggesting that LncPriCNet can achieve reliable and robust performance for different parameters.

### Case study

**Figure 3:**
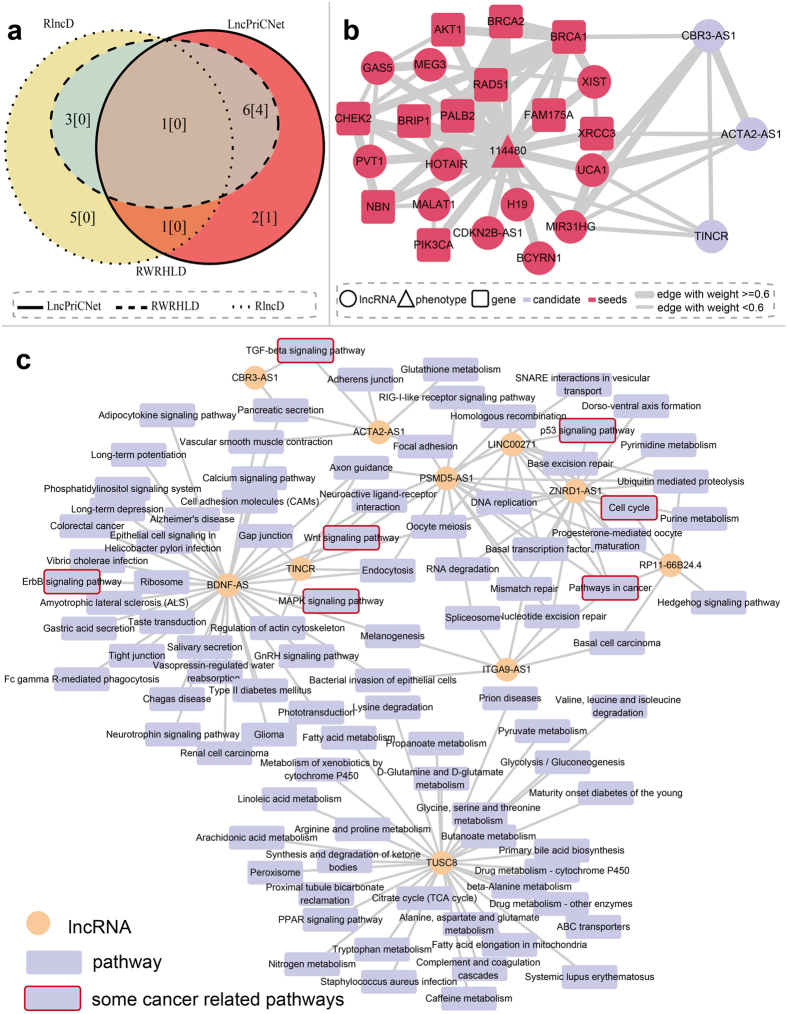
Case study, applying LncPriCNet to breast cancer. (**a**) Venn diagram of the top 10 ranked lncRNAs identified by LncPriCNet and two other methods. The numbers in square brackets denote lncRNAs with literature support. (**b**) The subnetwork of the top three lncRNAs (CBR3-AS1, TINCR and ACTA2-AS1) and seed nodes. (**c**) The network of the top 10 ranked lncRNAs and enriched pathways of their co-expressed genes.

Breast cancer is the leading cause of cancer mortality among women worldwide. We analyzed RNA-seq data consisting of 8 benign breast lesions, 8 ER-positive (ER+), 8 HER2-positive (HER2+), and 8 triple negative (TN) primary breast tumors (SRP019936). TopHat and cufflinks were used to align and assemble lncRNAs of each breast sample, and cuffdiff was used to identify differentially expressed lncRNAs for each type of breast cancer. We obtained 528 differentially expressed lncRNAs in ER+, HER+ and TN tumors. The breast cancer-related phenotype (MIM:114480), 11 known genes (BRCA2, PALB2, NBN, PIK3CA, RAD51, AKT1, CHEK2, XRCC3, BRCA1, BRIP1, FAM175A) and 11 known lncRNAs (BCYRN1, CDKN2B-AS1, GAS5, H19, HOTAIR, MIR31HG, MALAT1, MEG3, PVT1, UCA1, XIST) were used as seeds, and LncPriCNet, RWRHLD and RlncD were applied to score 528 differential lncRNAs. The top 10 lncRNAs for each method were predicted to be related to breast cancer, and their potential associations with breast cancer were investigated by manual literature mining (Table 1, Fig. 3a). The ranks of LncPriCNet and RWRHLD were similar, but they differed from the RlncD rank. Seven lncRNAs were ranked in the top 10 by both LncPriCNet and RWRHLD, and four of them had literature support (CBR3-AS1, ACTA2-AS1, TINCR, and TUSC8). Figure 3b illustrates the subnetwork of the top three lncRNAs and seed genes in LncPriCNet; the close connection indicated functional association in the same disease. CBR3-AS1 (PlncRNA-1), which was highly expressed in all three subtypes of breast cancer, was ranked first by LncPriCNet. It has been reported to be aberrantly expressed in both gastric cancer and prostate cancer, and silencing of CBR3-AS1 has been found to significantly reduce cell proliferation and induce apoptosis in prostate cancer cell lines^34^,^35^,^36^. High expression of ACTA2-AS1 (ZXF1) is related to a relatively poor prognosis and may promote invasion and metastasis in lung adenocarcinoma^37^. TINCR binds to staufen (STAU1) protein and mediates differentiated mRNA stabilization^38^. In addition, silencing TINCR expression inhibits cell proliferation, colony formation, tumorigenicity and apoptosis promotion^39^. TUSC8 ranked fourth and has been suggested to serve as an predictor for survival in cervical cancer^40^. Additionally, only LncPriCNet identified ZNRD1-AS1, which is involved in the occurrence and development of cancers by participating in the processes of DNA damage and repair and suppressing cell proliferation^41^,^42^,^43^.

To investigate the functional mechanism of the predicted breast lncRNAs, pathway enrichment analysis was performed. For each lncRNA, first, genes linked with the lncRNA (co-expression score above 0.6 between the lncRNA and mRNA) in our multi-level network were obtained. Then, Subpathway-GM^44^ was applied to perform pathway enrichment analyses (p-value < 0.01). Next, a lncRNA-pathway network was constructed for better visualization (Fig. 3c). The first-ranked lncRNA CBR3-AS1 was linked to the TGF-beta signaling pathway. TGF-beta has a suppressive effect in the early stage of tumorigenesis and hence is regarded as a tumor suppressor; it promotes tumor progression and metastasis during later stages^45^. In breast cancer, the TGF-beta signaling pathway promotes the metastasis of cancer via regulating the epithelial-to-mesenchymal transition (EMT)^46^. TGF-beta also may serve as a predictive and prognostic marker of cancer stage^47^. The fifth-ranked lncRNA, BDNF-AS, had the highest degree in the lncRNA-pathway network. It is involved in many breast cancer-related pathways, including the ErbB signaling pathway^48^,^49^, MAPK signaling pathway^50^, and wnt signaling pathway^51^. The term “pathway in cancer” received the highest degree among all pathways (degree = 5). Interestingly, five lncRNAs, ranked from fifth to tenth by lncPriCNet (LINC00271, ITGA9-AS1, PSMD5-AS1, RP11-66B24.4, ZNRD1-AS1), were directly linked to this pathway. Four of them did not have literature support, thus suggesting that our method can capture novel disease lncRNAs.

### A predicted landscape of disease-related lncRNAs

**Figure 4:**
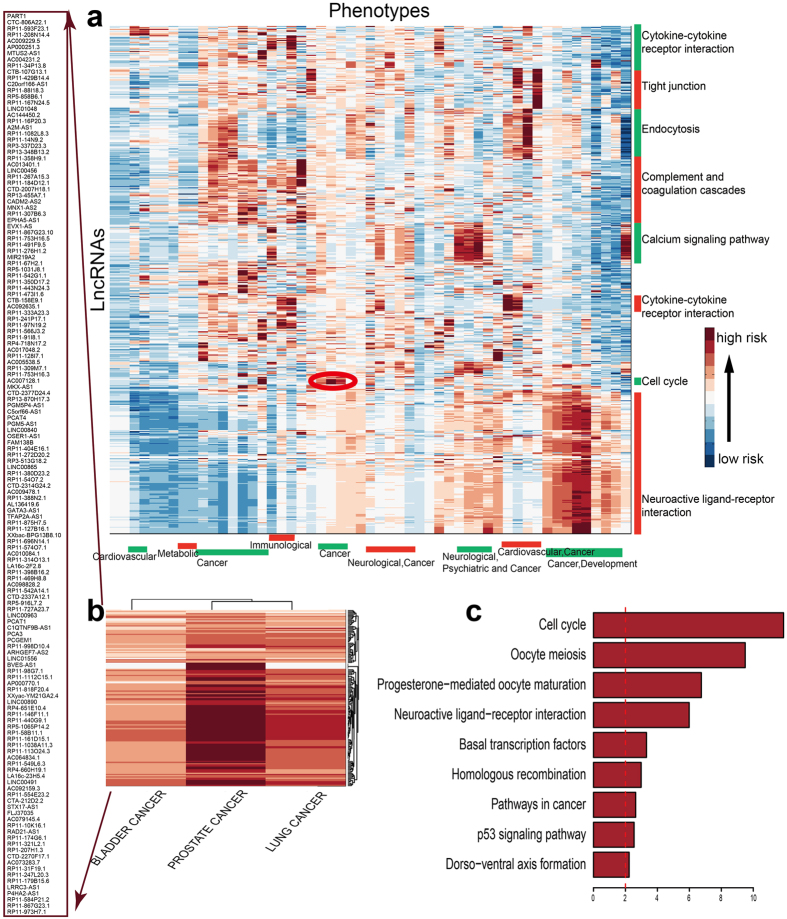
Global view of the predicted landscape of human disease lncRNAs. (**a**) Hierarchical clustering of the LncPriCNet scores between 53 phenotypes and 10082 lncRNAs. The color of each cell represents the LncPriCNet score of a lncRNA (row) for a phenotype (column). Phenotype clusters were annotated with enriched disease categories (bottom), and lncRNA clusters were annotated with the most enriched pathways of their co-expressed genes (right). The red circled region indicates a module composed of lncRNAs involved in the cell cycle process. (**b**) Zoom-in plot of the red circled region, involving 3 type of cancers and 156 high-risk lncRNAs. (**c**) Enriched pathways for the co-expressed genes of 156 high-risk lncRNAs.

We further used LncPriCNet to infer relationships between all lncRNAs and 53 disease phenotypes to chart a predicted lncRNA-disease landscape. First, scores between all 10082 lncRNAs and 53 disease phenotypes were computed to construct a score matrix. Then, a two-way hierarchical clustering method was used to reveal the organization of the human phenotype-lncRNA relationships (Fig. 4a). The phenotypes clustered together tended to have a similar molecular or genetic basis. Phenotype clusters were annotated with enriched disease classes. LncRNA clusters were annotated with the most enriched KEGG pathways of their co-expressed genes (Spearman correlation coefficient between lncRNA and gene higher than 0.6). The predicted disease-lncRNA landscape revealed some highly scored modules, each consisting of a set of functionally related lncRNAs implicated in a set of disease phenotypes. For instance, as illustrated in the red circle in Fig. 4a, a cancer-related module consisted of lncRNAs in the cell cycle pathway. From the zoomed-in plot in Fig. 4b, we found that this module included three types of cancer and 146 highly scored lncRNAs. Further inspection of the biological functions of these 146 lncRNAs showed that they were significantly enriched in cancer-related pathways, such as the cell cycle pathway, oocyte meiosis pathway and p53 signaling pathway (Fig. 4c). These cancers also shared three seed genes (H19, MALAT1 and MEG3). The similar molecular basis and high connection in the network may contribute to the modularity.

**Figure 5:**
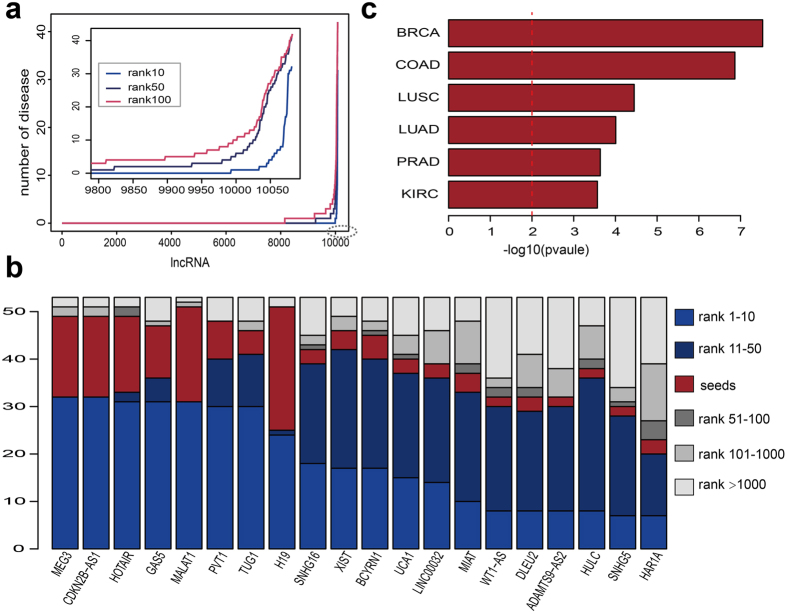
Statistics and analysis of lncRNA-disease landscape. (**a**) The number of disease phenotypes involving each lncRNA, with three different rank cutoffs. (**b**) The stacked plot of 20 lncRNAs, which are predicted to be associated with most phenotypes. (**c**) High-risk lncRNAs (top 50) are significantly overlapped with dysregulated lncRNAs in all six tumors (BRCA: Breast carcinoma; COAD: colon adenocarcinoma; LUSC: lung squamous cell carcinoma; LUAD: lung adenocarcinoma; PRAD: prostate adenocarcinoma; KIRC: kidney renal clear cell carcinoma).

Furthermore, we investigated the number of diseases in which each lncRNA was involved. Figure 5a shows that the majority of lncRNAs were not related to any disease, whereas some lncRNAs were related to multiple diseases. We extracted 20 lncRNA hotspots that were associated with most diseases and investigated their ranks in all 53 diseases (Fig. 5b). The highest-risk lncRNAs (MEG3 and CDKN2B-AS1) were involved in 32, 42, or 42 diseases when the top 10, 50, or 100 lncRNAs were regarded as disease-related. MEG3 is a maternally expressed imprinted gene that interacts with cAMP, p53, and GDF15 and modulates the activity of TGF-b genes by binding to distal regulatory elements, thereby playing a role in cell proliferation control^52^,^53^. Studies have reported that CDKN2B-AS1 is involved in various neoplasms^52^,^54^. In addition, we tested whether high-risk lncRNAs identified by LncPriCNet were more likely to be dysregulated in diseases. For this purpose, we downloaded the differentially expressed lncRNAs in six types of tumors^55^ and compared them with the top 50 lncRNAs ranked by LncPriCNet. As illustrated in Fig. 5c, high-risk lncRNAs significantly overlapped with dysregulated lncRNAs in all six tumors (Fisher’s Exact Test p-value < 0.01).

## Discussion

In this study, we present a novel computational method, LncPriCNet, to prioritize and predict disease candidate lncRNAs by integrating multi-level information regarding genes, lncRNAs, phenotypes and their associations. LncPriCNet showed clearly higher predictive power than previously published methods. More importantly, a breast cancer case study showed that LncPriCNet was able to capture well-documented lncRNAs as well as identify and infer the functional roles of novel lncRNAs. Furthermore, a human disease-lncRNA landscape revealed some phenotype modules with a similar molecular basis. The top 50 lncRNAs related to all 53 diseases were listed in (Supplementary Dataset 1). To further evaluate the predictions of the novel disease lncRNA, we also performed some literature research to the predicted top five diseae-related lncRNAs. For example, the top-ranked lncRNA PVT1 in hepatocellular carcinoma has been found to promote the proliferation and stem cell-like properties of hepatocellular carcinoma cells by stabilizing NOP2^56^ and might serve as a recurrence and supplementary diagnosis biomarker^57^,^58^. The top-ranked lncRNA HOTAIR in bladder cancer has been reported to be correlated with disease progression and may serve as a prognostic biomarker^59^,^60^. The serum levels of SNHG5, ranked fifth by LncPriCNet in melanoma, have been found to be significantly higher in patients with melanoma than in normal subjects and may serve as a new tumor marker of malignant melanoma^61^. Apart from cancer, the top-ranked lncRNA H19 associated with myocardial infarction has been reported to bind directly to miR-103/107 and regulate FADD expression and necrosis. The modulation of its levels may provide a new approach for preventing myocardial infarction^62^. LncRNAs GAS5, ranked fourth in atherosclerosis, is significantly increased in the plaques of atherosclerosis patients compared with normal subjects and may lead to new clinical applications^63^. LINC00929, which is significantly highly expressed in hereditary hemorrhagic disease^64^, was predicted as the top lncRNA by LncPriCNet. Furthermore, MALAT1, ranked second in Parkinson disease (PD), has been reported to show decreased expression in PD and to inhibit α-synuclein protein expression, thereby providing a neuroprotective effect in PD^65^. The fourth-ranked lncRNA in diabetes is MIAT, whose knockdown ameliorates diabetes mellitus-induced retinal microvascular dysfunction *in vivo* and inhibits endothelial cell proliferation, migration, and tube formation *in vitro*^66^. More literature citations supporting novel predictions are listed in Table S5. It is possible to select high-ranked lncRNAs from the prioritized list and test their causality through appropriate experiments. We also found disease lncRNA hotspots, such as MEG3 and CDKN2B-AS1, which have been reported to be involved in many diseases. Further analysis of six tumors showed that high-risk lncRNAs identified by LncPriCNet tend to be dysregulated in tumors, thus further supporting that LncPriCNet can identify disease-related LncRNAs.

The outstanding performance of LncPriCNet can be attributed to two aspects. First, LncPriCNet takes advantage of the multi-level information of the composite network. Abnormal phenotypes are usually a consequence of perturbed transcriptional levels, including not only coding genes but also lncRNAs. In addition, lncRNAs rarely perform biological functions alone but instead act as key regulators, such as scaffolds or sponges, that regulate genes^21^. The close connections of various levels may compensate for some missing information. For example, LncPriCNet performs well by using other information when information regarding known disease-associated lncRNAs is lacking. Second, LncPriCNet extends the RWR method and enables it to be used on a more complicated network model. It uses a global distance measure to prioritize candidate lncRNAs on the basis of their global similarity to known disease lncRNAs and genes, thereby capturing multi-level information of the composite network. This approach ensures that candidate lncRNAs are ranked according to the interaction information in the entire composite network rather than merely the local environment. However, there are still some limitations to this methodology. LncPriCNet relies on the topology of the composite network, and therefore the incompleteness and bias of the data may limit its performance. It will be improved when more accurate and complete resources are available.

Currently, the functions of most lncRNAs remain unknown, and the knowledge of disease-related lncRNAs are very limited. Candidate lncRNAs may be obtained by differential expression analysis or genome-wide association study analysis, but there are still too many candidates to experimentally validate. LncPriCNet prioritizes these candidate lncRNAs, allowing biologists to select high-ranking disease lncRNAs and test their functions. Furthermore, the lncRNA-disease and lncRNA-pathway network might provide clues as to the functional mechanisms of lncRNAs. Overall, LncPriCNet is a useful tool for disease lncRNA prioritization and provides better understanding of the molecular mechanisms of human disease at the lncRNA level, which may uncover new diagnostic and therapeutic opportunities. The strategy of the multi-level composite network could be used in other fields of biomedicine, such as disease, drug and target discovery.

## Additional Information

**How to cite this article**: Yao, Q. *et al*. Global Prioritizing Disease Candidate lncRNAs via a Multi-level Composite Network. *Sci. Rep.* **7**, 39516; doi: 10.1038/srep39516 (2017).

**Publisher's note:** Springer Nature remains neutral with regard to jurisdictional claims in published maps and institutional affiliations.

## Supplementary Material

Supplementary Information

Supplementary Dataset 1

## Figures and Tables

**Table 1:** Predicted breast cancer related lncRNAs, which were ranked in top 10 by LncPriCNet, RWRHLD or RlncD.

lncRNA	Rank (LncPriCNet)	Rank(RWRHLD)	Rank(RlncD)	Refereces
**CBR3-AS1**	**1**	**3**	97	34, 35, 36
**ACTA2-AS1**	**2**	**1**	31	37
**TINCR**	**3**	**2**	77	38,39
**TUSC8**	**4**	**4**	522	40,67,68
**BDNF-AS**	**5**	**5**	58	—
**LINC00271**	**6**	**6**	385	—
**ITGA9-AS1**	**7**	**10**	4	—
**PSMD5-AS1**	**8**	**18**	12	—
**RP11-66B24.4**	**9**	**11**	5	—
**ZNRD1-AS1**	**10**	**266**	266	41, 42, 43
**AC062029.1**	**20**	**9**	3	—
**LINC00900**	**22**	**7**	1	—
**LINC00638**	**23**	**8**	2	—
**LINC01004**	**27**	**14**	8	—
**C6orf3**	**31**	**12**	6	—
**RP4-583P15.10**	**42**	**16**	10	—
**LL0XNC01-116E7.2**	**44**	**13**	7	—
**MORF4L2-AS1**	**70**	**15**	9	—
