# Supplementary material for: Global Prioritizing Disease Candidate lncRNAs via a Multi-level Composite Network
Source: Sci Rep. 2017 Jan 4;7:39516. doi: 10.1038/srep39516 (PMC5209722; doi:10.1038/srep39516)
Supplement: Supplementary Information [file srep39516-s1.doc]

**Supporting information**

**Global Prioritizing Disease Candidate lncRNAs via a Multi-level Composite Network**

Qianlan Yao^1^, Leilei Wu^1^, Jia Li^2,3^, Li guang Yang^2,3^, Yidi Sun^2,3^, Zhen Li^1^, Sheng He^2,3^, Fangyoumin Feng^2,3^, Hong Li^2^,*, Yixue Li^1,2,4^*

^1^ School of Life Sciences and Biotechnology, Shanghai Jiao Tong University, Shanghai, 200031,China

^2^ CAS Key Laboratory for Computational Biology, CAS-MPG Partner Institute for Computational Biology, Shanghai Institute for Biological Sciences, Chinese Academy of Sciences, Shanghai, 200031, China

^3^ University of Chinese Academy of Sciences , Beijing, 100049, China

^4^ Collaborative Innovation Center of Genetics and Development, Fudan University, Shanghai 200433, China

* To whom correspondence should be addressed. Tel: 86-21-54920079; Fax: 86-21-54920079; Email: [yxli@sibs.ac.cn](mailto:yxli@sibs.ac.cn).

Correspondence may also be addressed to Hong Li. Tel: 86-21-54920079; Fax: 86-21-54920079; Email: lihong01@sibs.ac.cn

**Supplementary information**

**Table S1.** The statistic information of the multi-level composite network.

| Network | Number of nodes | Number of edges |
| --- | --- | --- |
| gene network | 9465 genes | 37039 |
| lncRNA network | 10082 lncRNAs | 50727383 |
| phenotype network | 4732 phenotypes | 18586 |
| gene-lncRNA association network | 10082 lncRNAs and 9465 genes | 95426130 |
| phenotype-gene association network | 1645 phenotypes and 1302 genes | 2043 |
| phenotype-lncRNA association network | 108 lncRNAs and 140 genes | 371 |

**Table S2**. The comparison of performance of LncPriCNet and two other methods in 10 disease classes. Gray shadow indicates disease classes in which the AUC value of LncPriCNet is higher than that of two other methods.

| DiseaseName | LncPriCNet | RWRHLD | RlncD |
| --- | --- | --- | --- |
| Endocrine* | 0.872 | 0.846 | 0.41 |
| Multiple* | 0.622 | 0.614 | 0.141 |
| Neurological* | 0.815 | 0.807 | 0.627 |
| AGING* | 0.955 | 0.953 | 0.951 |
| Cancer* | 0.945 | 0.943 | 0.536 |
| Cardiovascular* | 0.999 | 0.998 | 0.517 |
| Metabolic* | 1 | 1 | 0.647 |
| Psychiatric | 0.839 | 0.841 | 0.73 |
| Developmetal | 0.172 | 0.188 | 0.188 |
| Immunological | 0.749 | 0.785 | 0.738 |
| Mean* | 0.886857143 | 0.880142857 | 0.547 |
| AllDisease* | 0.933 | 0.926 | 0.543 |

**Table S3.** The effect of parameter of γ.

| $\delta$ | 0.1 | 0.3 | 0.5 | 0.7 | 0.9 |
| --- | --- | --- | --- | --- | --- |
| AUC | 0.943 | 0.941 | 0.936 | 0.933 | 0.928 |

**Table S4.** The effect of parameter of$\alpha$,$\beta$,$1\text{-}\alpha\text{-}\beta$,$x$,$\text{y}$ and $z$.

| ParaABC | case1 | case2 | case3 | ParaXYZ | Case4 | Case5 | Case6 |
| --- | --- | --- | --- | --- | --- | --- | --- |
| $\alpha$ | 0.1 | 0.1 | 0.8 | $x$ | 0.1 | 0.1 | 0.8 |
| $\beta$ | 0.1 | 0.8 | 0.1 | $y$ | 0.1 | 0.8 | 0.1 |
| $1\text{-}\alpha\text{-}\beta$ | 0.8 | 0.1 | 0.1 | $z$ | 0.8 | 0.1 | 0.1 |
| **AUC** | 0.927 | 0.936 | 0.931 | **AUC** | 0.936 | 0.909 | 0.879 |

**Table S5.** Some of literatures supporting novel predictions.

| Disease | LncRNA | Rank | Reference (pubmed id) |
| --- | --- | --- | --- |
| Bladder cancer | HOTAIR | 1 | 26800519;26469956;25030736;25994132 |
| Bladder cancer | GAS5 | 3 | 24069260;26548923 |
| Bladder cancer | PVT1 | 5 | [26517688](http://www.ncbi.nlm.nih.gov/pubmed/26517688) |
| MYOCARDIAL INFARCTION, | MALAT1 | 2 | [25035150](http://www.ncbi.nlm.nih.gov/pubmed/25035150) |
| DIABETES MELLITUS | MEG3 | 3 | [19966805](http://www.ncbi.nlm.nih.gov/pubmed/19966805); 26845358; 26603935 |
| DIABETES MELLITUS | MIAT | 4 | 25587098 |
| DIABETES MELLITUS | GAS5 | 5 | [26675493](http://www.ncbi.nlm.nih.gov/pubmed/26675493) |
| MYOCARDIAL INFARCTION | MALAT1 | 2 | [25035150](http://www.ncbi.nlm.nih.gov/pubmed/25035150) |
| HEPATOCELLULAR CARCINOMA | PVT1 | 1 | 25624916;27495068;25043274 |
| HEPATOCELLULAR CARCINOMA | SNHG16 | 2 | [27396952](http://www.ncbi.nlm.nih.gov/pubmed/27396952) |
| HEPATOCELLULAR CARCINOMA | UCA1 | 4 | 25760077;[26551349](http://www.ncbi.nlm.nih.gov/pubmed/26551349) |
| HEPATOCELLULAR CARCINOMA | TUG1 | 5 | 26336870;27339553 |
| PARKINSON DISEASE | NEAT1 | 1 | 27221610 |
| PARKINSON DISEASE | MALAT1 | 2 | [23756188](http://www.ncbi.nlm.nih.gov/pubmed/23756188); [27470562](http://www.ncbi.nlm.nih.gov/pubmed/27470562) |
| PARKINSON DISEASE | BDNF-AS | 3 | [23348013](http://www.ncbi.nlm.nih.gov/pubmed/23348013);[16402079](http://www.ncbi.nlm.nih.gov/pubmed/16402079) |
| PRADER-WILLI SYNDROME | MEG3 | 1 | 19800077;16906536 |
| TELANGIECTASIA, HEREDITARY HEMORRHAGIC | LINC00929 | 1 | [24603890](http://www.ncbi.nlm.nih.gov/pubmed/24603890) |
| AUTOIMMUNE DISEASE | H19 | 1 | [12937131](http://www.ncbi.nlm.nih.gov/pubmed/12937131) |
| SCHIZOPHRENIA | H19 | 2 | [20221451](http://www.ncbi.nlm.nih.gov/pubmed/20221451) |
| ATHEROSCLEROSIS | MIAT | 1 | 25587098 |
| ATHEROSCLEROSIS | MALAT1 | 3 | 25677520 |
| ATHEROSCLEROSIS | GAS5 | 4 | 27425867 |
| ATHEROSCLEROSIS | MIAT | 1 | 25587098 |
| ATHEROSCLEROSIS | MALAT1 | 3 | 25677520 |
| ATHEROSCLEROSIS | GAS5 | 4 | 27425867 |
| LEUKEMIA, CHRONIC LYMPHOCYTIC | PVT1 | 4 | [26010203](http://www.ncbi.nlm.nih.gov/pubmed/26010203) |
| LEUKEMIA, CHRONIC LYMPHOCYTIC | CDKN2B-AS1 | 5 | [23770605](http://www.ncbi.nlm.nih.gov/pubmed/23770605) |
| MELANOMA, CUTANEOUS MALIGNANT | SNHG5 | 5 | [26440365](http://www.ncbi.nlm.nih.gov/pubmed/26440365) |
| NASOPHARYNGEAL CARCINOMA | H19 | 1 | 27040767 |
| PROSTATE CANCER | XIST | 2 | [16261845](http://www.ncbi.nlm.nih.gov/pubmed/16261845) |
| PROSTATE CANCER | HOTAIR | 1 | 26411689 |
| THYROID CARCINOMA, PAPILLARY | HOTAIR | 3 | 27549736 |


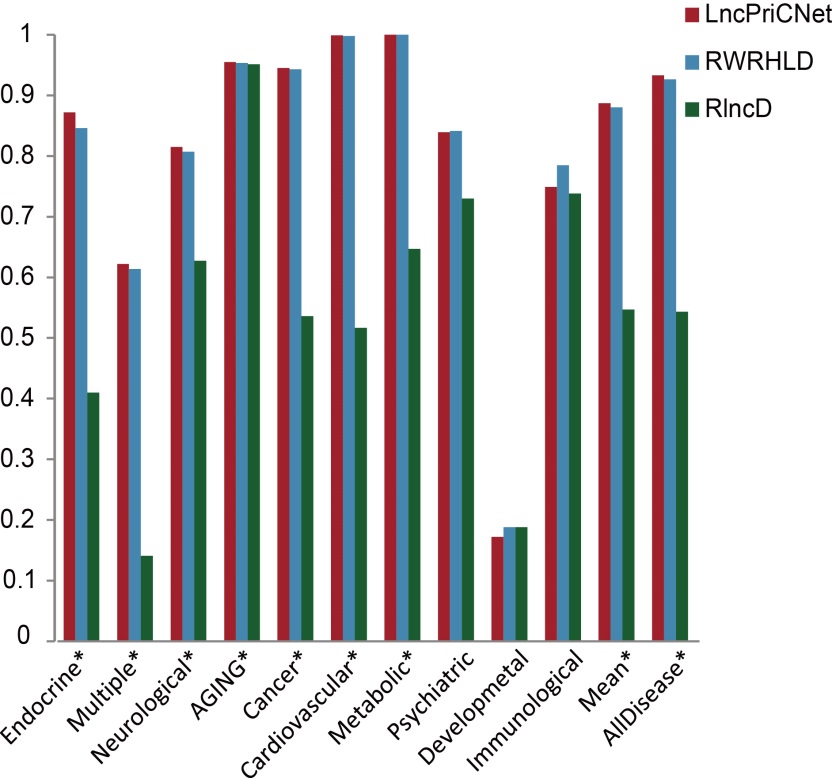


Figure S1. The comparison of performance of LncPriCNet and two other methods. Red bar indicates LncPriCNet; blue bar indicates RWRHLD and green bar indicates RlncD. Asterisk denotes the performance of LncPriCNet better than the other two methods
